# Fragmentation of tRNA in *Phytophthora infestans* asexual life cycle stages and during host plant infection

**DOI:** 10.1186/s12866-014-0308-1

**Published:** 2014-12-10

**Authors:** Anna KM Åsman, Ramesh R Vetukuri, Sultana N Jahan, Johan Fogelqvist, Pádraic Corcoran, Anna O Avrova, Stephen C Whisson, Christina Dixelius

**Affiliations:** Department of Plant Biology, Uppsala BioCenter, Linnéan Centre for Plant Biology, Swedish University of Agricultural Sciences, PO. Box 7080, SE-75007 Uppsala, Sweden; Cell and Molecular Sciences, The James Hutton Institute, Invergowrie Dundee, DD2 5DA UK; Current affiliation: Department of Evolutionary Biology, Uppsala University, SE-75236 Uppsala, Sweden

**Keywords:** Argonaute, Dicer, *Phytophthora infestans*, Potato, RNA silencing, Small RNA, tRF, tRNA

## Abstract

**Background:**

The oomycete *Phytophthora infestans* possesses active RNA silencing pathways, which presumably enable this plant pathogen to control the large numbers of transposable elements present in its 240 Mb genome. Small RNAs (sRNAs), central molecules in RNA silencing, are known to also play key roles in this organism, notably in regulation of critical effector genes needed for infection of its potato host.

**Results:**

To identify additional classes of sRNAs in oomycetes, we mapped deep sequencing reads to transfer RNAs (tRNAs) thereby revealing the presence of 19–40 nt tRNA-derived RNA fragments (tRFs). Northern blot analysis identified abundant tRFs corresponding to half tRNA molecules. Some tRFs accumulated differentially during infection, as seen by examining sRNAs sequenced from *P. infestans*-potato interaction libraries. The putative connection between tRF biogenesis and the canonical RNA silencing pathways was investigated by employing hairpin RNA-mediated RNAi to silence the genes encoding *P. infestans* Argonaute (PiAgo) and Dicer (PiDcl) endoribonucleases. By sRNA sequencing we show that tRF accumulation is PiDcl1-independent, while Northern hybridizations detected reduced levels of specific tRNA-derived species in the *PiAgo1* knockdown line.

**Conclusions:**

Our findings extend the sRNA diversity in oomycetes to include fragments derived from non-protein-coding RNA transcripts and identify tRFs with elevated levels during infection of potato by *P. infestans*.

**Electronic supplementary material:**

The online version of this article (doi:10.1186/s12866-014-0308-1) contains supplementary material, which is available to authorized users.

## Background

The mechanisms behind eukaryotic gene regulation have been extensively studied in animal, plant and fungal model organisms. Comparatively less is known about regulation of gene expression in heterokonts (stramenopiles), the eukaryotic group formed by diatoms, brown algae and oomycetes [1]. Members of the latter group resemble fungi in their morphology and lifestyle, but are phylogenetically only distantly related to true fungi [2,3]. The oomycetes encompass species living as saprophytes and pathogens of plants, insects, crustaceans, fish, and animals [4,5]. The most studied plant pathogenic oomycete is the potato late blight agent, *Phytophthora infestans* [6]. The *P. infestans* genome (~240 Mb) is one of the largest known within the genus *Phytophthora*, the majority comprising repetitive DNA [7]. The genome shows a bimodal organization pattern with densely packed core gene regions, interrupted by repeat-rich regions that are sparsely populated by genes [8]. A closer examination of the repeat-rich regions reveals enrichment for genes encoding disease-promoting effector proteins, which are at the forefront of evolution in this pathogen [9,10].

RNA silencing encompasses a set of mechanisms present in eukaryotes in which small RNAs (sRNAs) play central roles. It is the vanguard of genome defense against invasive nucleic acids such as transposons, viruses and transgenes [11,12]. Double-stranded RNA (dsRNA) from these myriad sources acts as triggers for gene silencing, initiating the degradation of complementary mRNA. This occurs via the generation of sRNAs by the class III ribonuclease (RNase) Dicer (Dcr; or Dicer-like, Dcl) and the association of the sRNA with Argonaute (Ago) family proteins. From the plethora of sRNA classes discovered and described, microRNAs (miRNAs), small interfering RNAs (siRNAs), and the Dcl-independent Piwi-interacting RNAs (piRNAs), are the most well characterized subtypes [12,13]. A class of sRNAs that was recently discovered through a number of deep sequencing studies is tRNA-derived RNA fragments (tRFs), 18–46 nt pieces derived from mature tRNA or the 3′ end of precursor-tRNA (pre-tRNA) [14-16]. tRNA fragmentation as a source of sRNA has been documented in organisms from all three domains of life [17].

Over the past decade, several compelling studies have shown that *P. infestans* possesses typical eukaryotic gene silencing pathways [18-22]. Silencing in *P. infestans* is reported to be functional at both the post-transcriptional and the transcriptional level, with sRNAs and heterochromatin formation acting to control transposons and transgenes [23-27]. Transcriptional silencing, and likely heterochromatin formation, has been shown to involve histone modification rather than cytosine methylation, and outward spread of silencing from heterochromatic loci has been detected at distances up to 600 bp [24-27]. Moreover, sRNAs are increasingly recognized as important players in plant-pathogen interactions. A recent report showed that pathogen-to-host sRNA transport resulted in silencing of host immunity genes and promotion of infection [28].

Here, in order to further identify and characterize the sRNA repertoire in *P. infestans*, the reads obtained through deep sequencing of sRNA were mapped to tRNAs. Data from two isolates and four developmental stages showed that the majority of tRFs mapped exclusively to the 5′ half of mature tRNA, had a guanosine at the 5′ end and mapped with the 3′ end in the tRNA anticodon loop region. Analysis of sRNA from infected potato leaves, on the other hand, identified a number of tRFs from *P. infestans* that were most abundant during infection, and the relative proportion of cleavage products from the 5′ and 3′ tRNA halves was found to shift during infection.

## Results and discussion

### sRNAs derived from *P. infestans* tRNAs

sRNA libraries were constructed from mycelia, sporangia, germinating sporangia and germinating cysts of two contrasting isolates (R0 and 3928A) via RNA-adapter mediated ligation. The two isolates differ in their specific virulence phenotypes and mating types: R0 is of the A1 mating type and is weakly pathogenic on potato [29], while 3928A belongs to the A2 mating type and is highly pathogenic [30]. The proportions of sequences derived from each life cycle stage are listed in Vetukuri et al. [21]. Here, the total sRNA dataset was mapped to tRNAs (Additional file 1: Table S1), revealing that less than 2% mapped to tRNA loci (1.9% in R0 and 1.5% in 3928A). Based on sequence identity (cut-off 90%), all tRNA and tRNA-like sequences from the *P. infestans* genome were sorted into clusters with the cd-hit algorithm [31], generating 230 clusters to which all the sRNA reads were mapped. Previously, deep sequencing from the different life cycle stages reported a skewed distribution of total read numbers [21]. A similar trend was observed for tRFs in this study, where more sRNA sequences mapped to tRNA in the sporangium stage compared to the mycelium stage.

**Figure 1:**
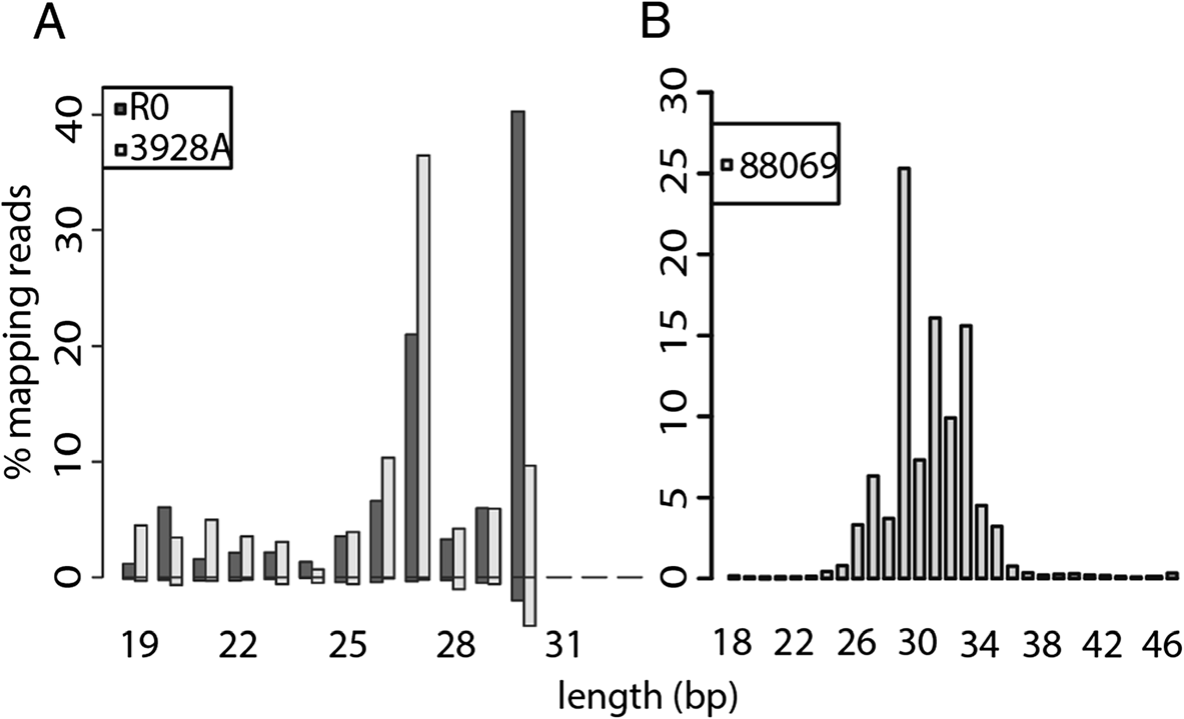
**Size distribution of sRNA reads mapping to tRNAs.** Shown are total sRNA reads from the mycelium life cycle stage of the three sequenced *P. infestans* isolates. **(A)** R0 and 3928A. **(B)** 88069. The percentages of sense and antisense reads are displayed on the positive and negative y-axes, respectively.

Mapping of sRNAs to individual tRNA clusters revealed that the majority of tRFs were 25–30 nt long and highly enriched for the sense strand, while the overall length distribution peaked at 27 and 30 nt (Figure 1A). Due to the bioinformatics pipeline applied to the SOLiD sequencing data, 30 nt was the upper read length limit.

### Most tRFs in *P. infestans* map to the 5′ end of mature tRNA

Studies in humans, plants and protists have revealed tRNA to be cleaved in the open loops of the RNA structure; in the anticodon-loop to generate tRNA halves and in the D- and T-loops to produce shorter tRFs, referred to as 5′ tRFs and 3′ CCA tRFs [15,16,32,33]. A fourth class of tRNA fragments, the 3′ U tRFs, is produced from the 3′ end of pre-tRNA and ends in the RNA polymerase III termination poly-U tract [15,16]. To establish which types of tRNA-derived fragments are present in *P. infestans*, we inspected the proportion of reads aligning to the respective ends of mature tRNA and the 3′ end of pre-tRNA, as well as the read lengths and inferred cleavage sites. Reads mapping with one end within the 5′ or 3′ terminal-most nucleotides of mature tRNA were considered as candidate 5′ or 3′ tRFs, respectively. On average 91% in R0 and 89% in 3928A were classified as 5′ tRFs, while only 4% and 3% were 3′ tRFs in R0 and 3928A, respectively (Table 1, Additional file 2: Figure S1, Additional file 3: Figure S2). Only three candidate 3′ U tRFs were found that started at the first nucleotide downstream of mature tRNA. Two had 3′ C while one had a single 3′ U, thus none ended in a poly-U tract. The finding of mostly 5′-mapping tRFs is comparable to the situation in *Trypanosoma cruzi*, where the overwhelming majority of nutritional stress-induced tRFs originate from the 5′ halves of mature tRNAs [34]. In short, the global profile of *P. infestans* tRFs indicates that these sRNAs originate from specific tRNA cleavage, whereby the 5′ fragments are favored for cellular retention.

**Table 1:** **Distribution of tRFs mapping to the 5′ and 3′ halves of mature tRNA in R0 and 3928A**

**Library**	**Number of reads**				**(%)**
	**5′**	**3′**	**Intermediate**	**Total**	**5′**
R0 germinating cysts	16130	1653	604	18387	88
R0 germinating sporangia	12669	314	771	13754	92
R0 mycelia	4704	118	402	5224	90
R0 sporangia	31177	776	1275	33228	94
3928A germinating cysts	13054	288	1021	14363	91
3928A germinating sporangia	12992	276	1015	14283	91
3928A mycelia	868	69	106	1043	83
3928A sporangia	10087	272	675	11034	91

### Abundant tRFs from tRNA Ile_cluster0

A high number of sRNA reads in the different life cycle stages and isolates mapped to the 5′ part of tRNA Ile_cluster0 (from now on termed as Ile0-5′tRFs). For example, 26% of the total tRFs identified in the R0 mycelia, and 25% of total tRFs in the 3928A germinating cysts mapped to this cluster (Additional file 1: Table S1). The number of reads mapping to tRNA Ile_cluster0 (anticodon AAU) was within the top five in all libraries except in the 3928A mycelia, which was also the library having the lowest number of total tRNA-mapping reads (Additional file 1: Table S1). An abundance of tRFs from a few specific tRNAs may suggest isolate- or life cycle specific isoacceptor preference, as was seen in *T. cruzi* [35]. In this protozoan parasite, tRFs were reported to derive primarily from tRNA^His^, tRNA^Arg^ and tRNA^Thr^, while in the diplomonad parasite *Giardia lamblia*, the most abundant tRFs during starvation-induced encystment derive from tRNA^Asp^ and tRNA^Gly^ [14]. We did not find that the overrepresentation of Ile0-5′tRFs was proportional to the numbers of tRNA^Ile^ (AAU) genes or the codon frequencies reported from two other *Phytophthora* species [36].

### Characteristics of tRF termini

**Figure 2:**
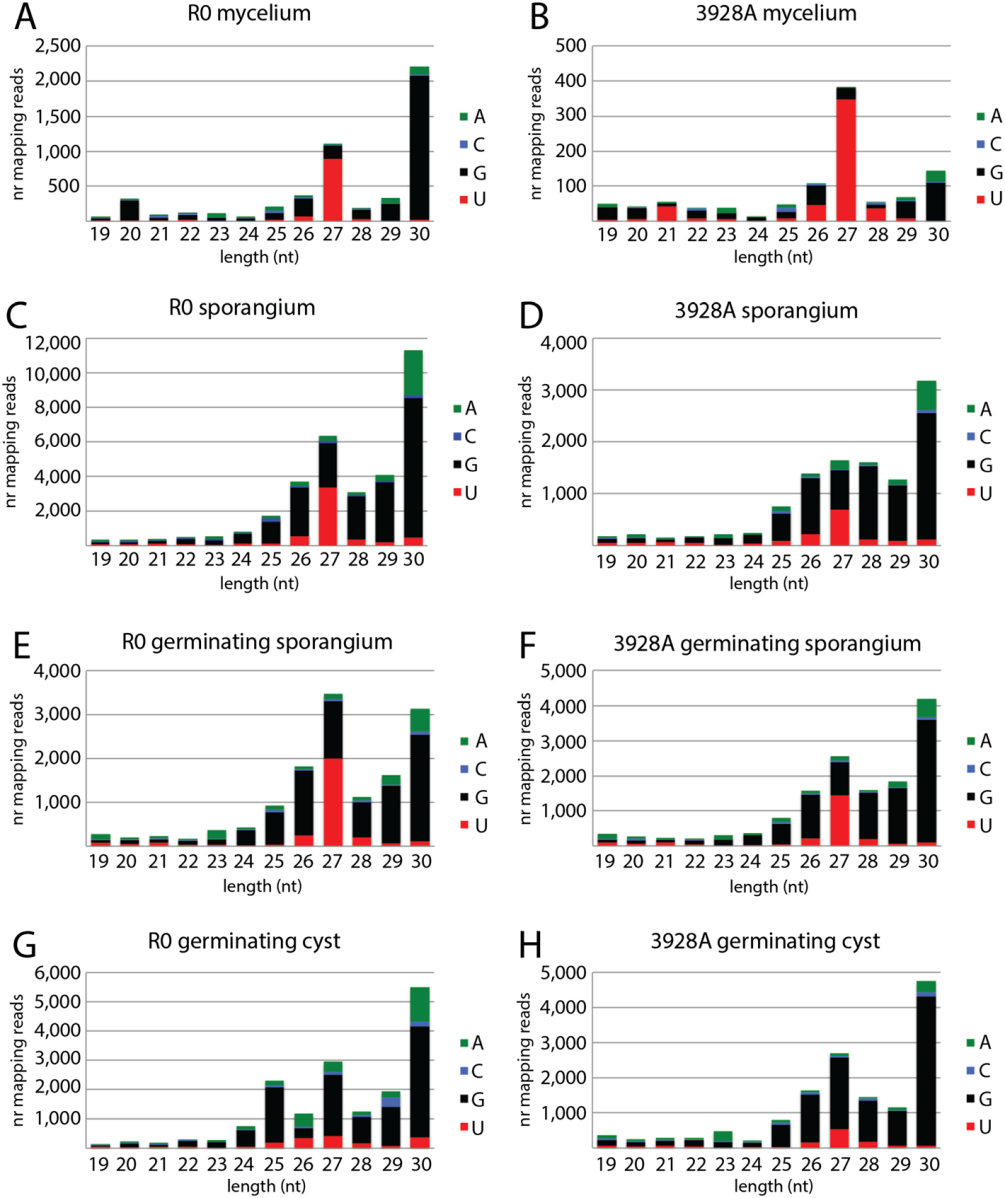
**The 5′ end nucleotide base identity of tRNA-mapping sRNAs in different life cycle stages. (A**, **C**, **E**, **G)** Isolate R0. **(B**, **D**, **F**, **H)** Isolate 3928A. Most tRF size classes started with 5′ G. The 27 nt sRNAs most frequently had 5′ U, except for in germinating cysts.

Organisms with multiple Ago protein homologs and sRNA classes have mechanisms for pairing of the sRNAs with the correct Ago protein [13]. In plants, this sorting is dictated by the sRNA 5′ terminal nucleotide, such that *Arabidopsis thaliana* Ago1 recruits mainly miRNAs, of which the majority begin with 5′ U [37]. *P. infestans* possesses four distinct *Ago* genes that are expressed throughout asexual development and during host infection [20]. PiAgo4 and PiAgo5 were shown to positively affect the accumulation of 32 nt long sRNAs from retrotransposons and other mRNAs, while 21 nt sRNAs from similar precursors were associated with PiDcl1 [21]. Nevertheless, any additional roles of the individual Agos in *P. infestans* gene silencing pathways have not been described. When inspecting the sRNA reads aligning to tRNA in the sequenced life cycle stages, an enrichment of 5′ terminal G was seen for tRFs of most length classes; on average 66% of all tRFs from the eight libraries had this particular 5′ nucleotide (Figure 2). 5′ tRFs comprised the majority of our sequencing libraries, thus this observation reflects the strong evolutionary conservation of the tRNA G_+1_ nucleotide, needed for RNaseP cleavage site recognition and successful pre-tRNA processing [38]. tRFs of 27 nt were an exception, since 5′ U was most prevalent in six out of the eight libraries (the two germinating cyst libraries were the exceptions). This suggests that 27 nt tRFs are generated by a different process and that they might be bound by a distinct PiAgo complex.

To form the mature amino acid-accepting 3′ end, a CCA trinucleotide is added post-transcriptionally to eukaryotic tRNA [39]. While addition of this terminal nucleotide modification is implicated in quality control [39,40], deacylation makes tRNA more prone to CCA loss [32]. We found very few reads ending in non-template CCA aligning to *P. infestans* 3′ tRFs. The 3′ CCA addition takes place before tRNA export from the nucleus and tRFs are cytoplasmic [16,39], so a step in the *P. infestans* 3′ tRF biogenesis pathway likely involves tRNA deacylation.

### Experimental validation of tRFs

**Figure 3:**
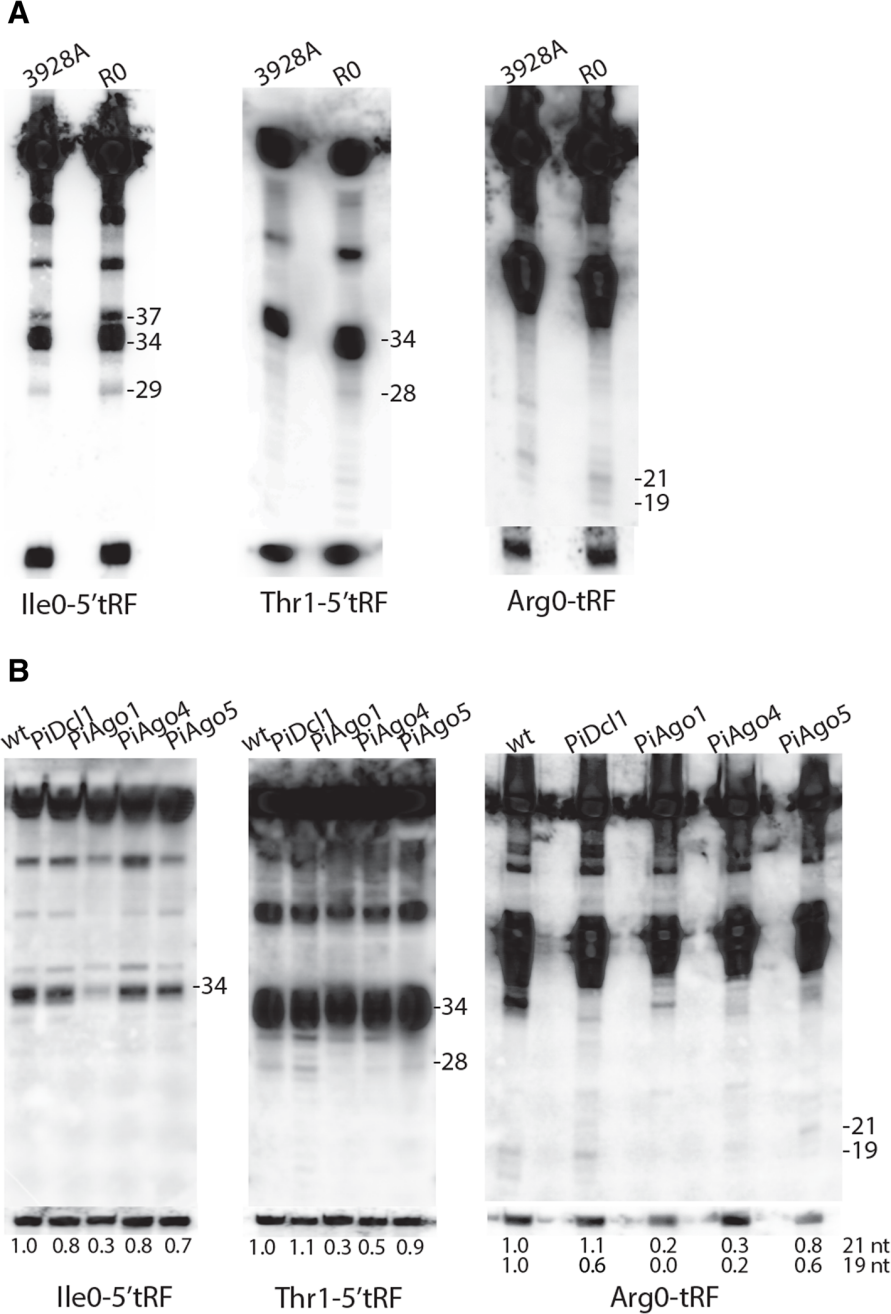
**Northern blot detection of sense sRNAs complementary to tRNA.** Hybridizations detected sense tRFs from tRNA Ile_cluster0, tRNA Thr_cluster1 and tRNA Arg_cluster0 in **(A)** wild-type (wt) isolates 3928A and R0, and in **(B)** wt isolate 88069 and transformant lines silenced for *PiDcl1*, *PiAgo1*, *PiAgo4*, *PiAgo5*. Approximate sizes in nucleotides are indicated to the right of each blot. The membranes were re-probed for 5S rRNA to control for equal loading of sRNAs (bottom). The signals in **(B)** were quantified, and values relative to the wt and normalized to 5S rRNA, are shown below each blot.

**Figure 4:**
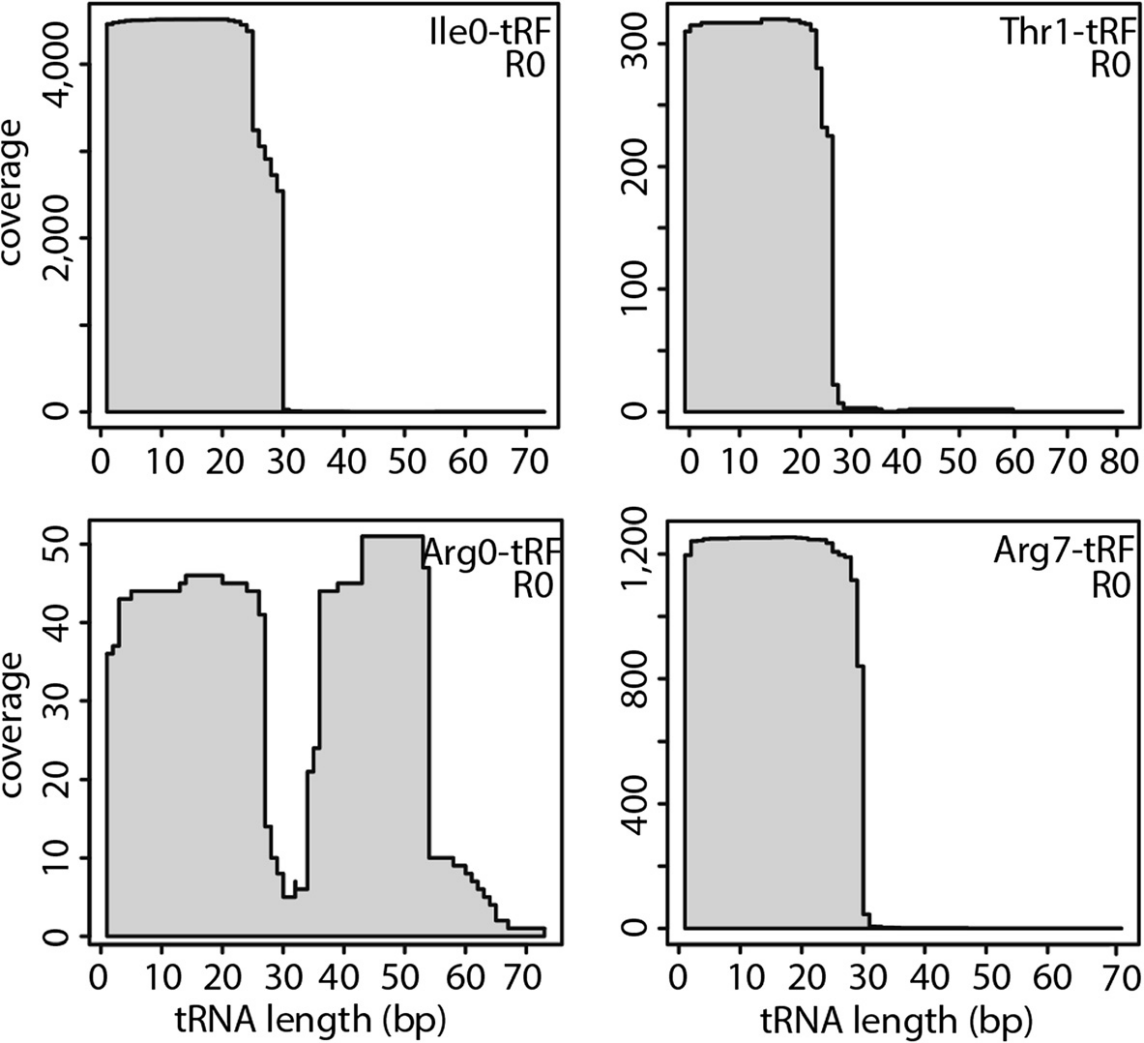
**Sequence read coverage at tRNA clusters.** sRNA read counts mapped along tRNA Ile_cluster0, tRNA Thr_cluster1, tRNA Arg_cluster0 and Arg_cluster7 in isolate R0. The profile was very similar in isolate 3928A.

**Figure 5:**
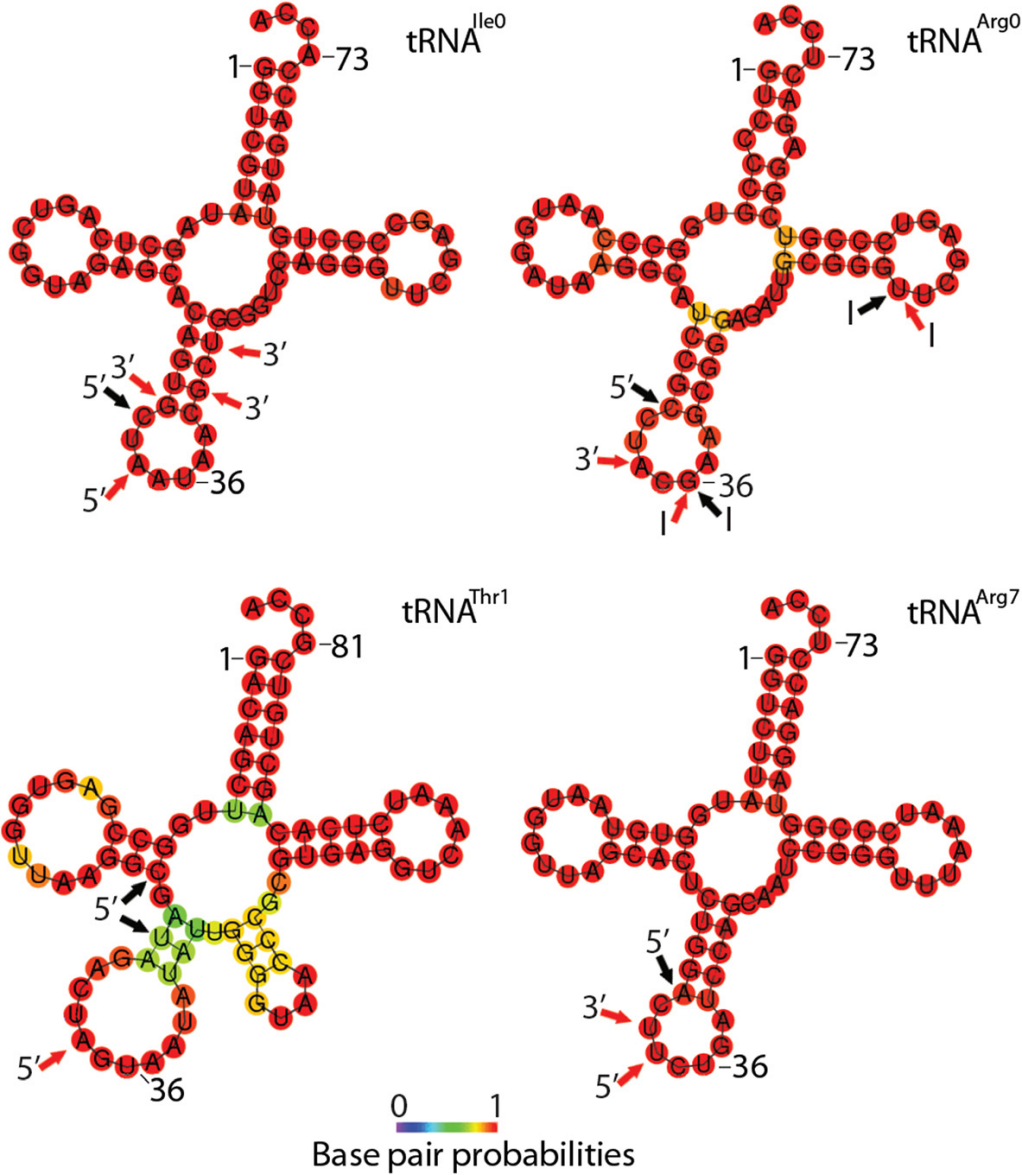
**Putative tRNA structures and predicted cleavage sites.** The predicted secondary structures of tRNA Ile_cluster0, tRNA Arg_cluster0, tRNA Thr_cluster1 and tRNA Arg_cluster7 are shown. The color code depicts base pair probabilities. Black arrows show 5′ cleavage sites determined by sRNA sequencing while cleavage sites suggested from Northern hybridizations are shown by red arrows for 5′ tRFs, internal (I) tRFs or 3′ tRFs. The lengths of 3′ tRFs are calculated excluding the 3′ CCA.

To verify the presence of tRNA cleavage products in *P. infestans*, candidate tRFs were chosen for Northern blot analyses of sRNA extracted from sporulating mycelium. First, Ile0-5′ tRFs were analyzed. In line with the high number of sRNA sequencing reads from the 5′ fragment of tRNA Ile_cluster0 (Additional file 1: Table S1), strong hybridization signals corresponding to Ile0-5′ tRFs were detected (Figure 3A). The length of the most abundant product, 34 nt (tRNA halves), agreed with the SOLiD sequencing data (Figure 4), since 30 nt was the upper read length cut-off. To map the cleavage site positions in the mature tRNA cloverleaf structure, tRNA Ile_cluster0 was *in silico* folded using Vienna RNAfold [41] (Figure 5). Next, sRNAs from tRNA Thr_cluster1 (Thr1-5′ tRFs) were examined. The main tRFs sequenced from this tRNA cluster, at 28 nt long, could be confirmed, although the strongest signal came from half-sized tRNAs (Figures 3A and 4). Both tRNA Ile_cluster0 and tRNA Thr_cluster1 showed strong hybridization signals from 5′ half tRNA molecules. Indeed, fragments of sizes consistent with being tRNA halves were detected for all tRNAs tested by Northern hybridization (Figure 3, Additional file 4: Figure S3, Additional file 5: Figure S4). These results suggest anticodon loop cleavage (at position 32–38) to be common in *P. infestans*, in agreement with observations from *Tetrahymena thermophila* and yeast [32,42]. Moreover, the tRNA half lengths are consistent with the known mechanistic action of RNases that cleave tRNA in the open loops, such as RNases T1, T2 and A [43].

A distinct fragmentation pattern was seen from tRNA Arg_cluster0. Sequencing identified two classes of Arg0-tRFs, a 27 nt long 5′ tRF and a 19–21 nt fragment mapping between the tRNA anticodon loop and the T-loop (Figure 4). Probing specifically for the internal fragment confirmed the presence of 19 and 21 nt tRFs (Figure 3) as well as detecting 38–45 nt products (Additional file 4: Figure S3A). Similar-sized tRFs were detected using a probe designed to bind to the 3′ end of tRNA Arg_cluster0 (Additional file 4: Figure S3B). Sequenced Arg0 5′ fragments were 27 nt long, however tRNA half products of about 34 and 36 nt were experimentally detected from the 5′ end (Additional file 4: Figure S3C). In conclusion, three types of tRFs appeared to be generated from tRNA Arg_cluster0: 5′ and 3′ half molecules through cleavage in the anticodon loop and an internal fragment generated by cleavages in both the anticodon- and T-loops (Figure 5).

### Detection of 3′ tRF sequences

The majority of sequenced tRFs mapped to tRNA 5′ termini. This selective maintenance of one tRNA cleavage product over the other is analogous to the preferred cellular retention of siRNA and miRNA guide strands as opposed to their respective passenger and miRNA* strands. We next extended the analyses to include 3′ tRFs, performing Northern hybridizations on the same sporulating mycelium samples as the corresponding 5′ tRFs had been detected in. tRNA Ile_cluster0 was chosen as the first candidate, due to the ease of experimental detection of its 5′ tRFs. Despite the lack of reads from 3′ half Ile_cluster0 tRNAs in the SOLiD libraries and the Illumina mycelium library (Figure 4, Additional file 6: Figure S5), clear hybridization signals from 3′ tRFs were detectable (Additional file 4: Figure S3D). This finding was not a unique feature of this particular tRNA: no 3′ fragments were present from tRNA Arg_cluster7 in any of the sequencing libraries (Figure 4, Additional file 6: Figure S5) but fragments from both 5′ and 3′ tRNA halves were seen by Northern hybridization (Additional file 4: Figure S3E, F). This indicates that the cleavage products from both halves of the two tRNAs are maintained after anticodon loop processing, contradicting the results from sequencing.

### Changes in the tRF repertoire during host plant infection

To examine tRNA fragmentation during *P. infestans* infection of potato, sRNA libraries were generated from potato leaves infected with isolate 88069 and a *Dcl-*silenced transformant (*PiDcl1*), sampled at three time points: 24, 48 and 72 hours post inoculation (hpi). These, and control samples from the mycelium life cycle stage, were sequenced using the Illumina HiSeq 2500 platform. Mapping of sRNA reads to the tRNA clusters revealed major length classes of 29, 31 and 33 nt, highly enriched for sense reads (Figure 1B). As in the SOLiD data, a bias was seen towards the tRNA 5′ end, although the over-representation was not as large (Tables 1 and 2, Additional file 7: Figure S6). The fraction of 5′ tRFs in both 88069 and the *PiDcl1*-silenced line was somewhat higher at the three infection time points (on average 71% and 79%, respectively) than in mycelium (55% and 49%). A more dramatic change in the relative production of 5′ and 3′ tRNA halves was observed in *T. cruzi*, where under nutritional stress 98% of the tRFs derived from 5′ halves [34], while 87% were processed from 3′ halves in cells differentiated into the host-infective life cycle stage [44]. Differences in the proportions of 5′ mapping tRFs were observed between the isolates used in our study (90% in R0, 83% in 3928A and 55% in 88069). It should be noted that the lower proportion revealed from 88069 was from sequencing using Illumina technology while SOLiD was used for the R0 and 3928A isolates. It is therefore difficult to associate the observed differences in tRF abundance with any pathogenicity characteristics.

**Table 2:** **Distribution of tRFs mapping to the 5′ and 3′ halves of mature tRNA in 88069 and the** ***PiDcl1*** **silenced mutant**

**Library**	**Number of reads**				**(%)**
	**5′**	**3′**	**Intermediate**	**Total**	**5′**
88069 24 h	9909	3740	698	14347	69
88069 48 h	13130	2932	1573	17635	74
88069 72 h	29752	8601	4067	42420	70
88069 mycelia	37540	22957	8300	68797	55
PiDcl1 24 h	5365	1059	443	6867	78
PiDcl1 48 h	8389	2032	680	11101	76
PiDcl1 72 h	3302	317	343	3962	83
PiDcl1 mycelia	75783	52973	25937	154693	49

Despite the absence of sRNA sequencing reads derived from the 3′ ends of tRNA Ile_cluster0 and tRNA Arg_cluster7, 3′ tRFs were readily detected by Northern hybridization. This discrepancy might be due to sequencing bias, which is common in sRNA sequencing studies [45]. Factors that influence the process of cDNA construction, such as 5′ and 3′ nucleotide identities and modifications, in addition to sRNA secondary structure, could have biased our sequencing result towards 5′ tRFs. Similarly, different sequencing protocols vary in their sensitivity to the mentioned RNA features [45], making it difficult to do strict comparisons of data generated by different sequencing platforms. This could underlie the observed higher proportion of 5′ tRFs from the SOLiD-sequenced isolates (R0 and 3928A) compared to the Illumina-sequenced material (88069 and *PiDcl1*).

The Illumina sequencing data confirmed the 5′ tRNA halves observed by Northern hybridization from tRNA Ile_cluster0 and tRNA Arg_cluster7 as well as both the 5′ and the 3′ halves from tRNA Arg_cluster0. In contrast, only trace amounts of the 19 nt internal tRF from tRNA Arg_cluster0 were seen (Additional file 6: Figure S5 and Additional file 8: Figure S7). Cleavage products from Ile tRNA_cluster0 were abundant in the datasets from both sequencing platforms (Additional file 1: Table S1, Additional file 9: Table S2). The most prominent product from this tRNA was the 5′ tRNA half, as detected by both Northern hybridization and Illumina sequencing (Figure 3, Additional file 6: Figure S5). Notably, the sequence size distribution was very narrow at the mycelium stage, showing a sharp peak at 32 nt, while no single dominant size class was observable at 24 and 72 hpi (Additional file 8: Figure S7). Since half tRNA-sized Ile0-5′ tRFs were the most mycelium-enriched of all tRFs (Additional file 9: Table S2 and Additional file 10: Figure S8), this suggests a reduction in the number of Ile_cluster0 tRNAs that are cleaved in the anticodon loop during infection.

### Differential tRF abundance during infection progression

To compare the tRF read counts in-between the different time points and with the sample from cultured mycelium, heat maps were generated from the normalized read counts from each tRNA cluster. A number of tRFs in isolate 88069 showed elevated read numbers in one or more of the three infection-stage libraries. We chose to focus on the top twenty tRFs most abundant across the four libraries (Additional file 10: Figure S8). Both the second-most infection-responsive tRF (Pro1-tRF, 31 nt; Additional file 8: Figure S7) and the third-most infection-responsive tRF (Leu6-tRF, 26 nt) peaked at the biotrophic stage of the interaction (24 hpi). This phase is characterized by plant mesophyll tissue invasion by *P. infestans* intercellular hyphae that acquire nutrients from the host [46]. The same two tRFs were shown to decrease at the transition stage (48 hpi) and decline further at necrotrophy (72 hpi). At this point the plant tissue was extensively colonized and sporulating lesions were formed on the leaf surface. Lys3-tRFs (25, 26 nt), the most elevated tRF under infection, peaked at this necrotrophic stage. A third tRF accumulation profile was seen for Cys1-tRF, Glu5-tRF, Asp0-tRF and Gly1-tRF, which peaked at 48 hpi (Additional file 10: Figure S8).

The first function ascribed to tRNA cleavage products was in regulation of various stress responses [47], a characteristic shared by organisms such as fungi (yeast), protists (*Giardia*, *Tetrahymena*), animals (mammals, flies) and plants (*Arabidopsis*) [14,32,42,48]. It is premature to infer a specific role for tRNA cleavage in the *P. infestans*-host interaction, but we note that: (1) a number of tRNA cleavage products accumulate at comparably higher levels during infection than in mycelium, and (2) tRFs accumulate differentially at the three infection time points. Possibly, some tRNAs or tRFs may be involved in the biotrophic-to-necrotrophic infection stage progression. Although further evidence is needed, it could be speculated that regulated tRNA fragmentation serves to decrease the levels of specific tRNAs, such as tRNA Pro_cluster1, for which the cleavage product was the most abundant tRF at 24 and 48 hpi. This mechanism could for example be part of a stress-induced translational re-programming response. It is notable that proline has been proposed to be an osmoregulator controlling zoospore discharge in *P. nicotianae* [49], and to regulate the osmotic pressure needed for penetration of the potato leaf cuticle in *P. infestans* [50]. The levels of free proline in the latter study were four times higher in the pre-infection life cycle stage compared to mycelium. The differential levels of Pro_cluster1 tRFs may reflect a mechanism used by *P. infestans* to regulate proline levels in the cell. The narrow size distribution of Pro1-tRFs and their accumulation pattern exclusively from the tRNA 5′ half, argue against the notion of Pro1-tRFs as random degradation products (Additional file 6: Figure S5, Additional file 8: Figure S7). Fragmentation of tRNA is typically not a feature of the well-characterized eukaryotic tRNA turnover pathways, which act from the tRNA 5′ and 3′ ends [39].

### Analysis of PiDcl and PiAgo involvement in tRF biogenesis

It is being increasingly realized that tRFs associate with canonical RNAi components such as Dcl and Ago proteins in different organisms. In humans, studies of the interactions between hAgo3 and hAgo4 with 20–22 nt tRFs identified a tRF class formed through Dcl processing that is capable of *trans*-silencing of target sequences, similar to siRNAs and miRNAs [51]. In stressed *Drosophila* cells, tRNAs and tRFs compete with dsRNA for processing by the siRNA-specific Dcl (Dcr-2) [48]. Based on these previous studies and on the known, conserved roles of Dcl and Ago proteins in sRNA-binding and processing, we hypothesized that silencing *PiDcl1* and the four *PiAgos* would lead to perturbed tRF levels in *P. infestans*. Constructs targeting *PiDcl1*, *PiAgo1*, *PiAgo3*, *PiAgo4* and *PiAgo5* were designed and stable hairpin-mediated gene silencing transformations were successful with all except *PiAgo3*. After analysis of the levels of transcript knockdown in the generated independent transformant lines by quantitative Reverse Transcription PCR (qRT-PCR), the most silenced line for each gene was chosen for further studies (Additional file 11: Figure S9). Silencing of PiDcl1 and PiAgos to similar levels has previously been shown to impact on sRNA accumulation and functionality of silencing [20,21].

As seen by Northern blotting for nine different tRFs (Figure 3B, Additional file 5: Figure S4F-J), their production and stability was not negatively influenced by *PiDcl1* knockdown, in contrast to findings in flies and mammals [48,52]. This was further confirmed by Illumina sequencing from *PiDcl1*, as no major differences in tRF levels or sizes were seen compared to the wild type. For illustration, the size distribution of Ile0-tRFs in 88069 and the *PiDcl1*-silenced line is shown in Additional file 8: Figure S7; similar results were found for other tRFs that were analyzed. We note that a role for PiDcl proteins in tRNA fragmentation cannot completely be ruled out, since a second Dcl protein (PiDcl2) was identified in the *P. infestans* genomic sequence trace archive after initiation of this study [22].

Reduced tRF levels were repeatedly observed in the *PiAgo1*-silenced line (e.g. Ile0-5′ tRF, Trp2-5′ tRF, Ser6-5′ tRF), and to a lesser extent in the *PiAgo4*-silenced line (Thr1-5′tRF, Arg0-tRF; Figure 3B). Fragments affected by *PiAgo1* knockdown were not homogenous in size, reduced signals being observed for short tRFs (19 and 21 nt), as well as for 26–30 nt tRFs and for half-sized tRNA fragments. Levels of some 5′ halves were unperturbed in the *PiAgo1*-silenced line (tRNA Glu_cluster4, tRNA Leu_cluster0), suggesting that *P. infestans* has more than one tRNA fragmentation pathway. No decrease in tRF levels was seen in the *PiAgo5-*silenced line. This might be due to the low degree of gene silencing in this transformant (Additional file 11: Figure S9).

Ago proteins are specialized RNA binding proteins with preference for short RNAs such as miRNAs, siRNAs and piRNAs [53]. Crystallographic studies have shown that the PAZ domain accommodates the sRNA 3′ hydroxyl group, while the 5′ phosphate binding pocket resides within the MID domain. The structures and lengths of tRNAs and their cleavage products are distinct from those of canonical Ago-interacting sRNAs, making the formation of Ago-tRF complexes appear intriguing. Yet, new functions of Ago proteins continue to be discovered, and different Ago homologs vary in their ability to bind structured RNAs [53-55]. Couvillion and co-workers [56] proposed a model for the mechanism of Twi12-tRF complex formation in *Tetrahymena*, wherein stacking of the tRNA T-loop and acceptor stems creates a binding substrate of suitable size for Piwi recognition. Tight binding of the tRNA acceptor stem 3′ side would in this case make the 5′ endless protected from nucleases, explaining why 3′ tRFs are predominately loaded onto Twi12. It is possible that binding of PiAgo1 to tRNA similarly dictates the asymmetric accumulation of *P. infestans* tRFs preferentially from the tRNA 5′ side. The internal Arg0-tRF was also shown to be PiAgo1-dependent. This indicates that additional binding partners are involved in the putative PiAgo1-tRF complex, or that there are multiple PiAgo1-tRF binding configurations.

### Crosstalk between RNA silencing and tRNA cleavage pathways

At least two alternative roles for PiAgo1 in the tRNA cleavage pathways can be envisaged. On one hand, it is possible that PiAgo1 plays a direct role in tRF biogenesis, by endonuclease cleavage of tRNAs into 5′ and 3′ fragments. Alternatively, the protein might act at a step after tRNA cleavage, binding and stabilizing tRFs produced by another, as-yet unknown, nuclease. No convincing evidence for Ago-mediated tRNA cleavage has been put forward to date, thus the second possibility appears more likely. Besides Dcl and Ago proteins, the *P. infestans* genome encodes several additional classes of endoribonucleases. Two well-studied eukaryotic tRNA cleavage nucleases are yeast Rny1p (RNase T2 type) and mammalian Angiogenin (RNase A type) [17,57,58]. Since the RNase A superfamily is vertebrate-specific [59], we speculate that the *P. infestans* tRNA cleavage nuclease is of the RNase T2 class, of which at least five predicted proteins are encoded by the organism’s genome (PITG_11433, PITG_16015, PITG_01495, PITG_15217 and PITG_08597). PiAgo1 is critical for maintaining gene silencing in *P. infestans* [20], which implies that bound tRFs have the potential to regulate RNA silencing through competition with other sRNAs for PiAgo1 loading. The tRNA cleavage products would then classify as “competing endogenous RNAs”, regulatory RNAs competing for shared molecular targets and regulating each other through RNA-RNA crosstalk [60].

## Conclusions

Deep sequencing is a powerful tool for sRNA research. Biases inherent in protocols and chemistries used in different platforms are however inevitable [45]. With this in mind, we conclude that this study identified a number of tRFs in the stramenopile *P. infestans*. By sRNA sequencing and Northern blot analysis in three isolates, we show that tRFs accumulate from both 5′ and 3′ halves of mature tRNA. According to sequencing data, 5′ tRFs were more abundant than 3′ tRFs under both asexual development and infection of host tissue, whereas the relative proportion of 5′ fragments was elevated during host infection. Overall, a number of tRFs were identified that seem more abundant during plant infection, possibly regulating pathogenicity related functions, analogous to recent findings of sRNAs as key players in host-pathogen interactions. Finally, knockdown of the genes encoding *P. infestans* Dcl and Ago proteins suggested an involvement of PiAgo1 in the tRF pathways. This crosstalk with *P. infestans* canonical RNA silencing pathways might lead to competition with other classes of sRNAs for PiAgo1 binding, and suggests a mechanism through which tRFs could regulate the activities of the RNA silencing machinery.

## Methods

### *P. infestans* culturing and transformation

sRNA preparations from *P. infestans* wild type isolates R0, 3928A and 88069 and from a *PiDcl1*-silenced transformant were used for deep sequencing. Culturing conditions, preparation of life cycle stage samples [21], infections and leaf sample collection (88069 and *PiDcl1*, potato cultivar Desirée) were as previously described [20]. The methods for designing inverted repeat silencing constructs, generation of stable *P. infestans* transformants and their maintenance were as for earlier reported transformants [21]. Primers used for cloning from *P. infestans* DNA are listed in the same study.

### Quantitative reverse transcription PCR analysis

Extraction of total RNA, cDNA synthesis, qRT-PCR, and subsequent data analysis were carried out as outlined by Vetukuri et al. [20] including primer sequences. Transcript levels were normalized to the internal control *PiActA* (AAA33749) and presented as the fold change relative to the calibrator sample (88069 mycelium).

### sRNA sequencing and computational analysis

Total RNA was extracted using the mirVana™ miRNA isolation Kit (Ambion). For SOLiD sequencing, the methods for RNA library preparation, deep sequencing and data analysis were as reported [21], except that the sRNA read mapping was done with increased stringency (read length 19–30 nt). Illumina library preparation was done with the Illumina TruSeq small RNA sample preparation kit and the sequencing reactions were run on a HiSeq 2500 platform at SciLifeLab (Stockholm, Sweden). All Illumina adaptor molecules were first filtered out from the sRNA sequences, and reads shorter than 18 bases or having less than 5 bp adaptor sequence were excluded from further analysis. The sRNA reads were aligned to the *P. infestans* genome tRNA dataset (http://www.broadinstitute.org) using bowtie2 v2.1.0 [61] by first clustering the tRNA sequences with cd-hit [31], using 90% identity as a threshold for assignment to a cluster. From the clusters, a consensus sequence was constructed using the most common base at each position. Recording of sRNA lengths, numbers, starting base and sense/antisense reads was done using SAMtools v0.1.19 [62] and custom python scripts. The counts of reads mapping to each of the tRNA clusters were obtained using the BEDTools intersect command [63], and normalized using the R package DESeq [64]. Heatplots were generated from the normalized read counts at each tRNA cluster using the gplots package (cran.r-project.org/web/packages/gplots/) in the statistical software package R v3.0.2. Unless otherwise stated, all calculations were performed in R (http://www.R-project.org) [65].

### tRNA secondary structure prediction

The Vienna RNAfold webserver was used for prediction of tRNA cloverleaf secondary structures (http://rna.tbi.univie.ac.at) [41]. Default settings were used, except for changing the folding temperature to 20°C to reflect the optimal growth temperature of *P. infestans* and not allowing the CCA end to form any base pairs*.*

### sRNA Northern hybridization

Low-molecular-weight fraction RNA was extracted from sporulating mycelium and analyzed by Northern hybridization [21] using DNA oligonucleotides 5′ end-labeled with γ-^32^P-ATP (Additional file 12: Table S3). A probe complementary to *P. infestans* 5S rRNA was used for the loading control. Quantification of bands was done with the program QuantityOne (BioRad) and normalized to the loading control.

## Availability of supporting data

The SOLiD sequencing data sets supporting the results of this article are available in NCBI’s Gene Expression Omnibus (GEO) repository, accession GSE62674 (http://www.ncbi.nlm.nih.gov/geo/query/acc.cgi?acc=GSE62674). The Illumina sequencing data sets are available in the GEO database, accession GSE63292 (http://www.ncbi.nlm.nih.gov/geo/query/acc.cgi?acc=GSE63292).

## Additional files

Additional file 1: Table S1. SOLiD sRNA sequencing reads mapped to tRNA clusters in *P. infestans* isolates R0 and 3928A. Four life cycle stage samples were analyzed from each isolate. Additional file 2: Figure S1. Proportion of reads mapping to either the 5′ or the 3′ end of tRNA (see labels at right). Four sequenced life cycle stages in R0. Additional file 3: Figure S2. Proportion of reads mapping to either the 5′ or the 3′ end of tRNA (see labels at right). Four sequenced life cycle stages in 3928A. Additional file 4: Figure S3. Detection of 5′ and 3′ half tRNAs. Northern hybridization in wild type isolates and the *PiDcl1* silenced line. **(A, B)** 3′ half tRNAs from tRNA Arg_cluster0. **(C)** 5′ half tRNAs from tRNA Arg_cluster0. **(D)** 3′ half tRNAs from tRNA Ile_cluster0. **(E)** 5′ half tRNAs from tRNA Arg_cluster7. **(F)** 3′ half tRNAs from tRNA Arg_cluster7. Approximate sizes in nucleotides are indicated to the right of each blot. Shown below each tRF Northern blot is the same membrane re-probed for 5S rRNA to control for equal loading. The signals in **(A)** were quantified and the value in *PiDcl1* is shown to the right of the blot, relative to the wild type (wt) 88069 and normalized to 5S rRNA. Additional file 5: Figure S4. Detection of 5′ half tRNAs in wild type isolates and the *PiDcl1* silenced line. Northern hybridization in **(A-E)** isolates R0 and 3928A and **(F-J)** isolate 88069 and the *PiDcl1* silenced line. **(A)** tRNA Met_cluster3. **(B)** tRNA Asn_cluster1. **(C)** tRNA Asp_cluster0. **(D)** tRNA Arg_cluster4. **(E)** tRNA Gly_cluster0. **(F)** tRNA Glu_cluster4. **(G)** tRNA Arg_cluster7. **(H)** tRNA Leu_cluster0. **(I)** tRNA Trp_cluster2. **(J)** tRNA Ser_cluster6. Approximate sizes in nucleotides are indicated to the right of each blot. Shown below each tRF Northern blot is the same membrane re-probed for 5S rRNA to control for equal loading of sRNAs. The signals in **(F-J)** were quantified and values in *PiDcl1* are shown to the right of the blot, relative to the wild type (wt) 88069 and normalized to 5S rRNA. Additional file 6: Figure S5. Sequence read coverage at tRNA clusters. sRNA read counts mapped along tRNA Ile_cluster0, tRNA Arg_cluster7, tRNA Arg_cluster0, and tRNA Pro_cluster1 in the mycelium life cycle stage library and the three infection stage libraries in isolate 88069. The y-axis in each graph represents the total tRF read count. Additional file 7: Figure S6. Proportion of reads mapping to either the 5′ or the 3′ end of tRNA (see labels at right). The mycelium life cycle stage (myc) and three infection stage time points (24, 48 and 72 hpi) in isolate 88069. Additional file 8: Figure S7. Size distribution of Illumina sRNA reads mapping to individual tRNA clusters. sRNA sequence read lengths in the three infection stage libraries and the mycelium life cycle stage library in isolate 88069 and in the *PiDcl1* silenced mutant. Y-axis: the percentage mapped sRNA reads of each length class. Additional file 9: Table S2. Illumina sRNA sequencing reads mapped to tRNA clusters in isolate 88069 and the *PiDcl1* silenced transformant. Three infection stage samples (24, 48 and 72 hpi) and one mycelium stage sample were analyzed from each line. Additional file 10: Figure S8. Heatmap displaying tRNA-mapping read numbers. The top 20 tRFs exhibiting the highest numbers of normalized read counts across the four sequenced libraries in isolate 88069. 24, 48 and 72 hpi; three infection time points, myc; mycelium. Color key: normalized read counts. Additional file 11: Figure S9. Transcript levels in hairpin-transformed lines assayed by real-time RT-PCR. The four silenced lines used for Northern blot analysis of tRF levels. mRNA levels were normalized to the *ActinA* (*PiActA*) reference gene and are presented relative to the wild type isolate 88069 (assigned a value of 1.0). Total numbers of individual lines analyzed per gene: *PiDcl1-* 10, *PiAgo1-* 8, *PiAgo4*- 10 and *PiAgo5*- 6. Error bars represent confidence intervals from two technical replicates per sample in the PCR reaction. Additional file 12: Table S3: Sequences of oligonucleotides used as DNA probes.

## Abbreviations

Ago: Argonaute
Dcl: Dicer-like
hpi: Hours post inoculation
sRNA: Small RNA
tRF: tRNA-derived RNA fragment
