# Supplementary figures and images for: Fragmentation of tRNA in Phytophthora infestans asexual life cycle stages and during host plant infection
Source: BMC Microbiol. 2014 Dec 10;14:308. doi: 10.1186/s12866-014-0308-1 (PMC4272539; doi:10.1186/s12866-014-0308-1)

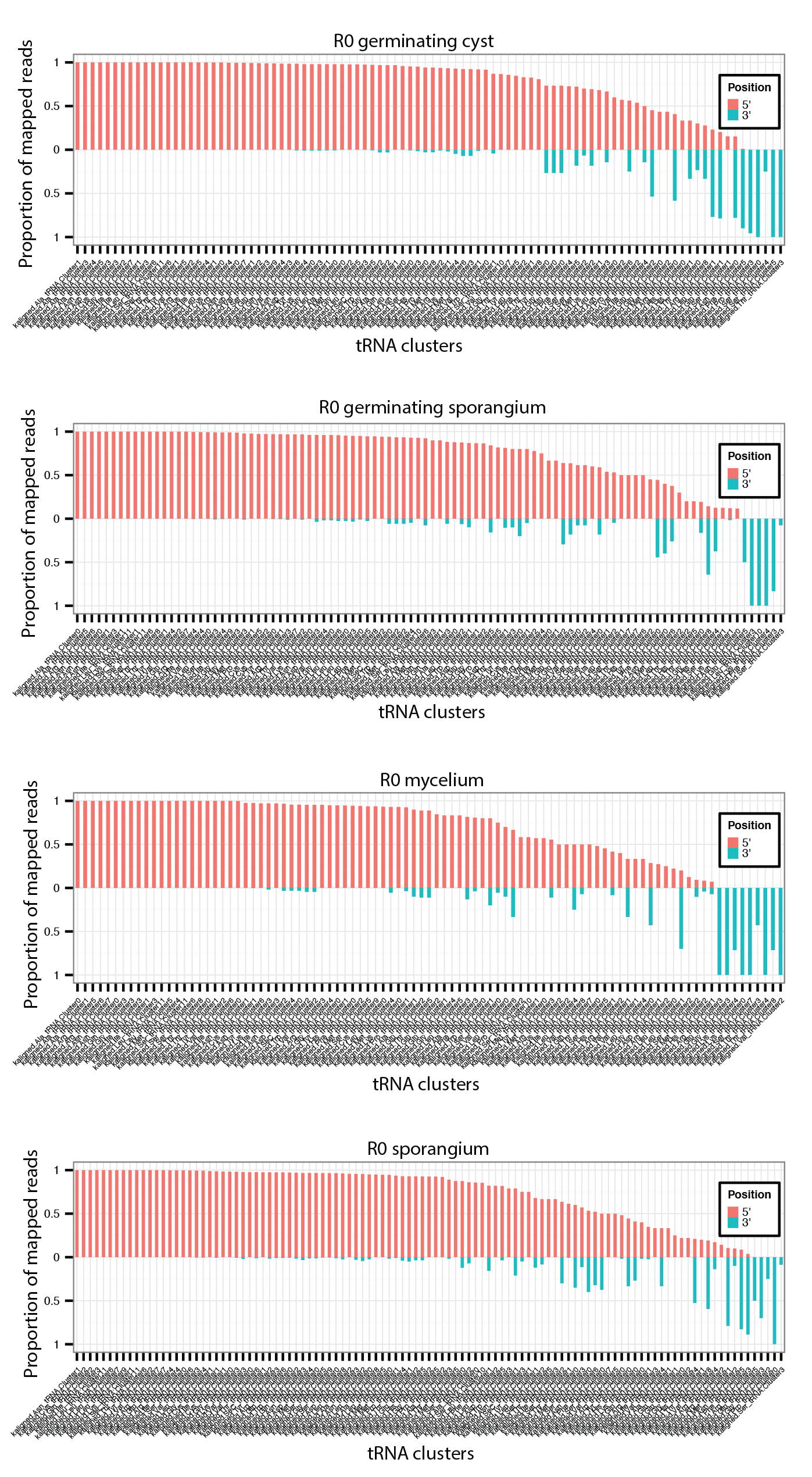

Supplement: Additional file 2: Figure S1. — Proportion of reads mapping to either the 5′ or the 3′ end of tRNA (see labels at right). Four sequenced life cycle stages in R0. [file 12866_2014_308_MOESM2_ESM.tiff]

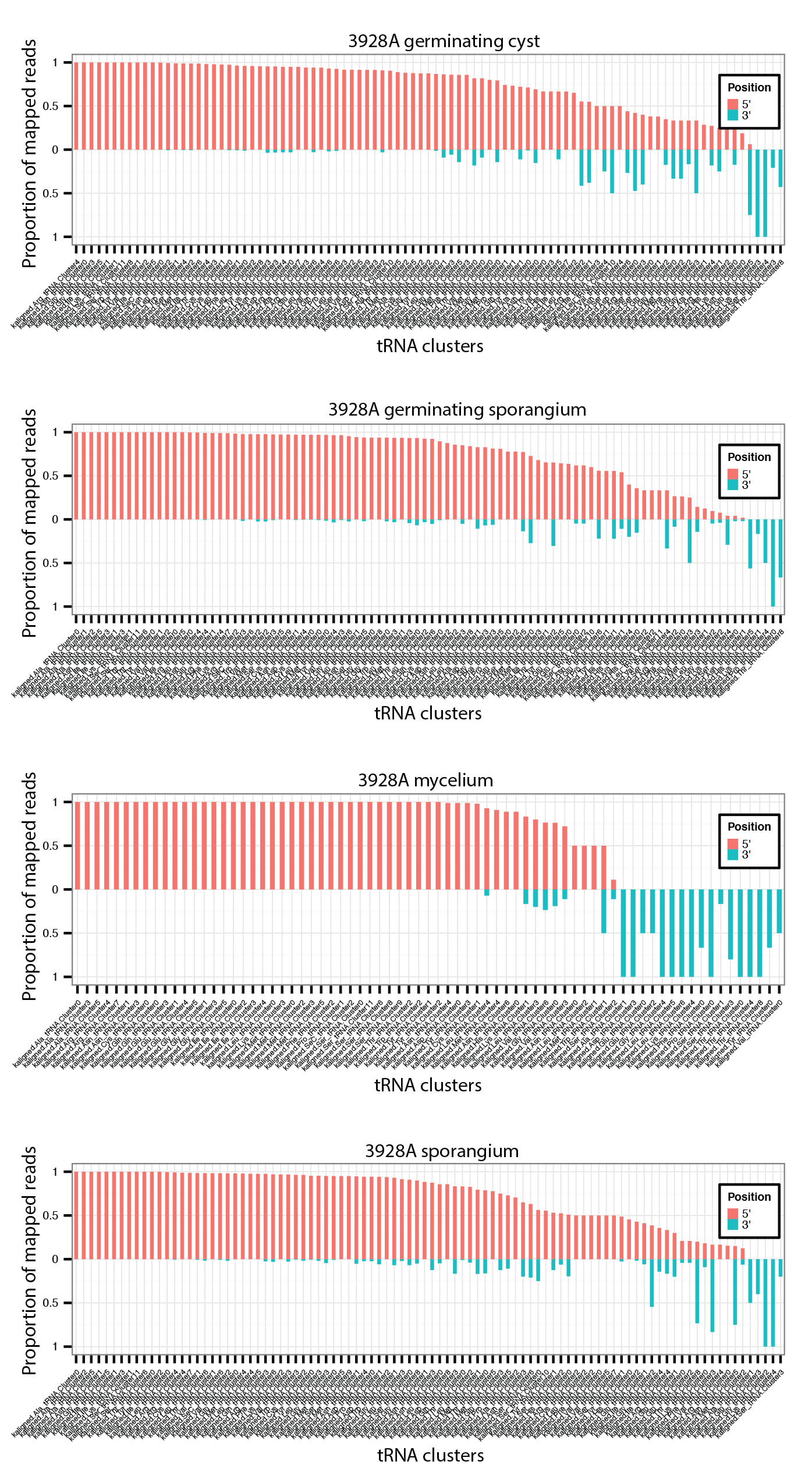

Supplement: Additional file 3: Figure S2. — Proportion of reads mapping to either the 5′ or the 3′ end of tRNA (see labels at right). Four sequenced life cycle stages in 3928A. [file 12866_2014_308_MOESM3_ESM.tiff]

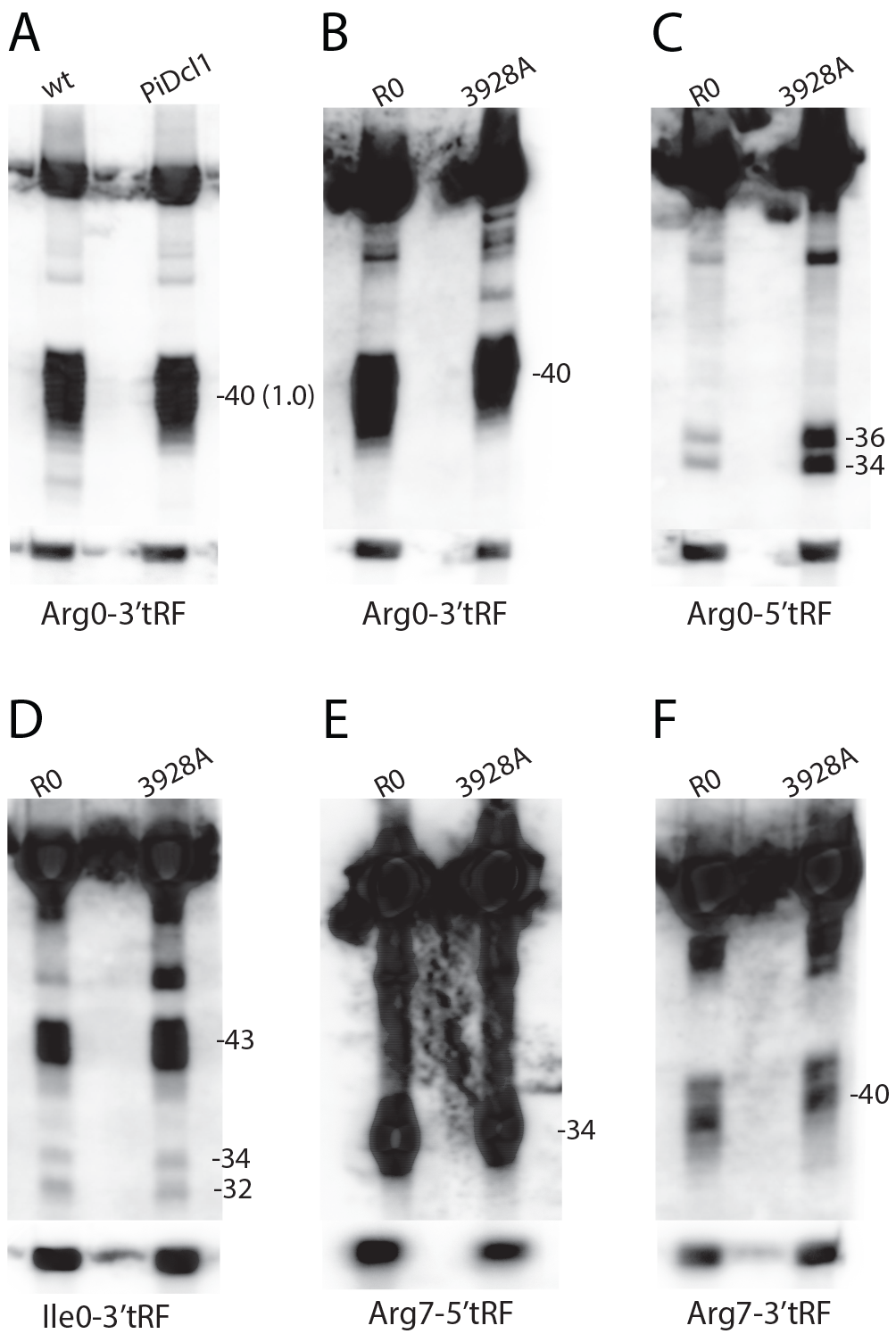

Supplement: Additional file 4: Figure S3. — Detection of 5′ and 3′ half tRNAs. Northern hybridization in wild type isolates and the PiDcl1 silenced line. (A, B) 3′ half tRNAs from tRNA Arg_cluster0. (C) 5′ half tRNAs from tRNA Arg_cluster0. (D) 3′ half tRNAs from tRNA Ile_cluster0. (E) 5′ half tRNAs from tRNA Arg_cluster7. (F) 3′ half tRNAs from tRNA Arg_cluster7. Approximate sizes in nucleotides are indicated to the right of each blot. Shown below each tRF Northern blot is the same membrane re-probed for 5S rRNA to control for equal loading. The signals in (A) were quantified and the value in PiDcl1 is shown to the right of the blot, relative to the wild type (wt) 88069 and normalized to 5S rRNA. [file 12866_2014_308_MOESM4_ESM.tiff]

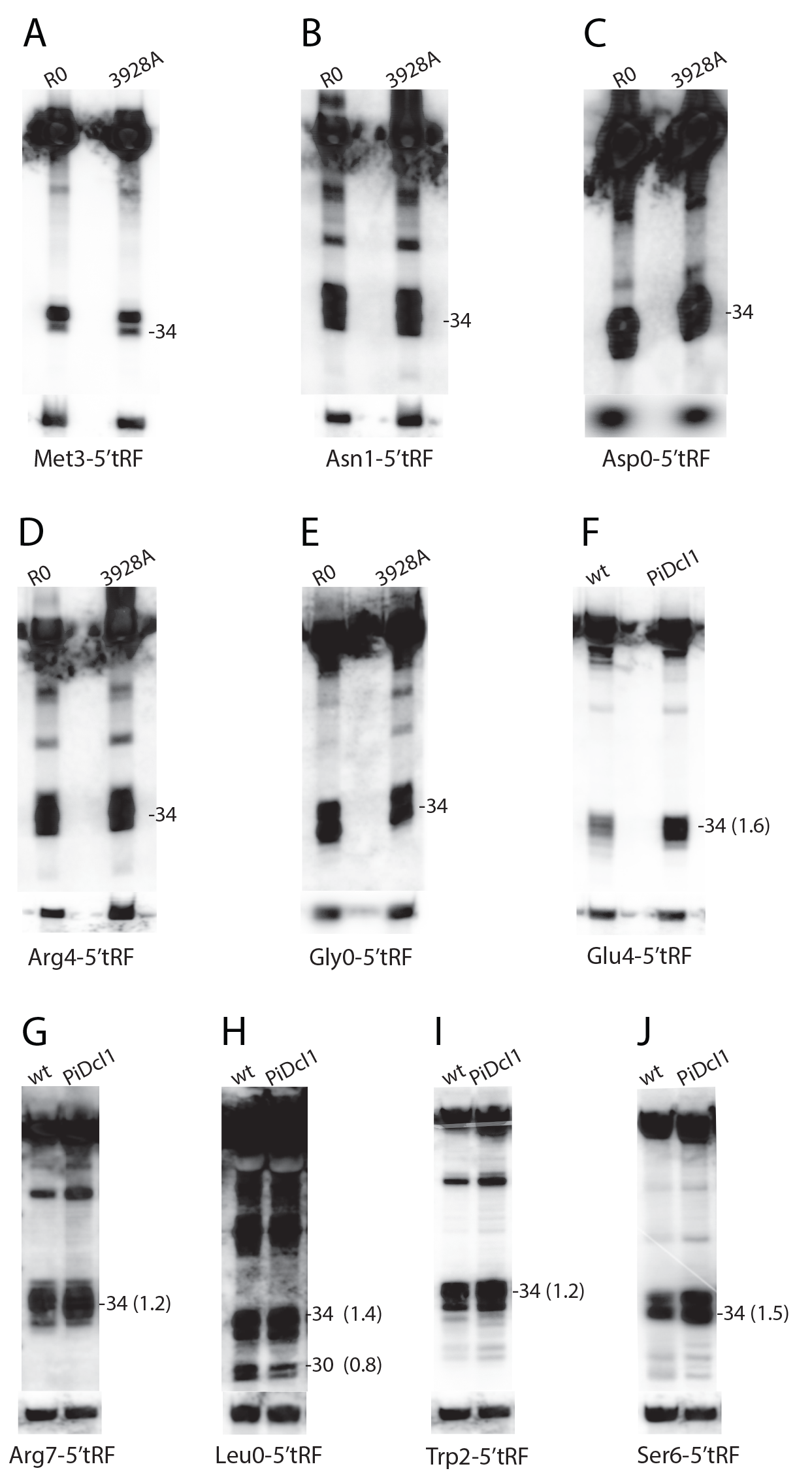

Supplement: Additional file 5: Figure S4. — Detection of 5′ half tRNAs in wild type isolates and the PiDcl1 silenced line. Northern hybridization in (A-E) isolates R0 and 3928A and (F-J) isolate 88069 and the PiDcl1 silenced line. (A) tRNA Met_cluster3. (B) tRNA Asn_cluster1. (C) tRNA Asp_cluster0. (D) tRNA Arg_cluster4. (E) tRNA Gly_cluster0. (F) tRNA Glu_cluster4. (G) tRNA Arg_cluster7. (H) tRNA Leu_cluster0. (I) tRNA Trp_cluster2. (J) tRNA Ser_cluster6. Approximate sizes in nucleotides are indicated to the right of each blot. Shown below each tRF Northern blot is the same membrane re-probed for 5S rRNA to control for equal loading of sRNAs. The signals in (F-J) were quantified and values in PiDcl1 are shown to the right of the blot, relative to the wild type (wt) 88069 and normalized to 5S rRNA. [file 12866_2014_308_MOESM5_ESM.tiff]

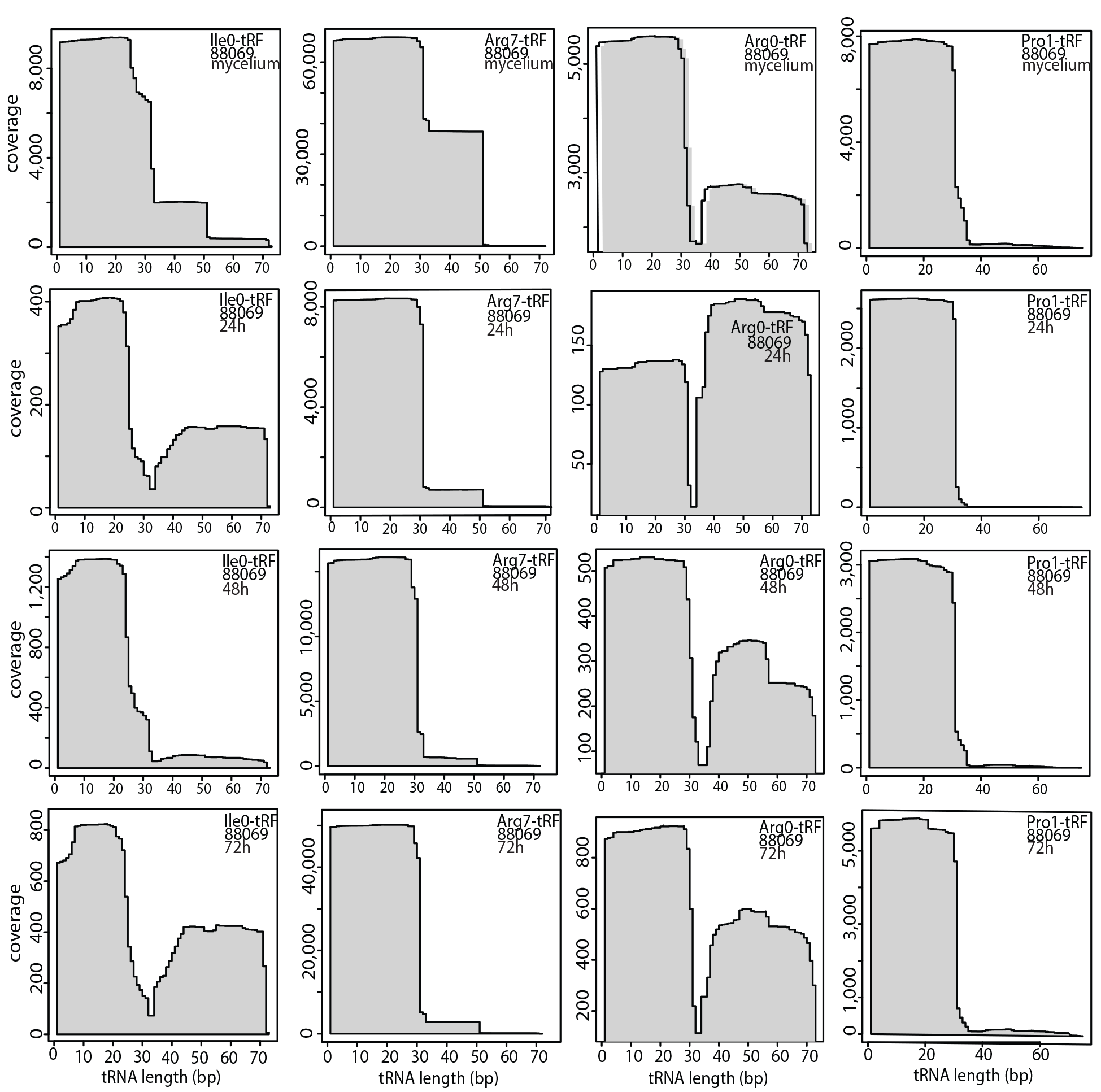

Supplement: Additional file 6: Figure S5. — Sequence read coverage at tRNA clusters. sRNA read counts mapped along tRNA Ile_cluster0, tRNA Arg_cluster7, tRNA Arg_cluster0, and tRNA Pro_cluster1 in the mycelium life cycle stage library and the three infection stage libraries in isolate 88069. The y-axis in each graph represents the total tRF read count. [file 12866_2014_308_MOESM6_ESM.tiff]

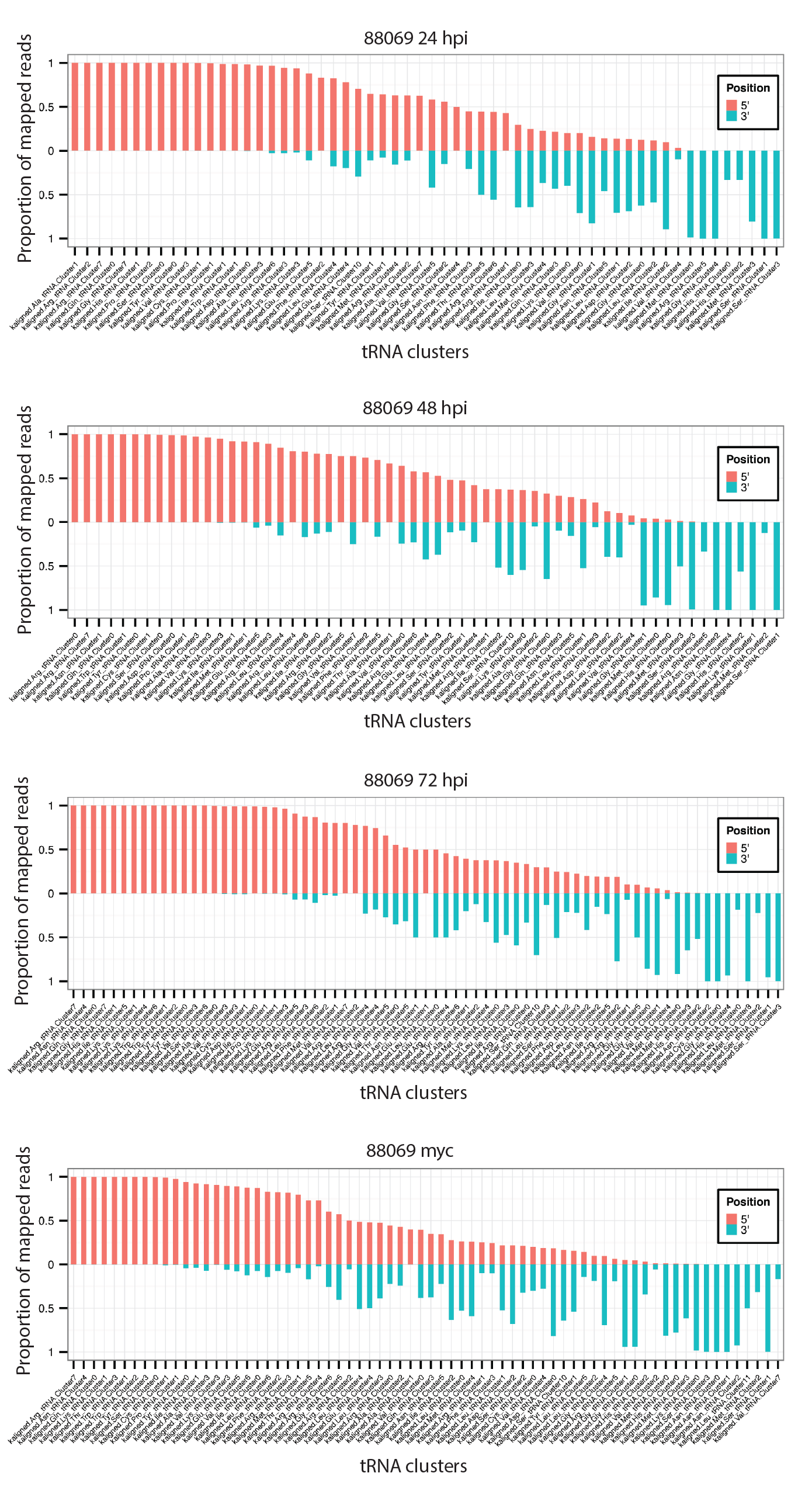

Supplement: Additional file 7: Figure S6. — Proportion of reads mapping to either the 5′ or the 3′ end of tRNA (see labels at right). The mycelium life cycle stage (myc) and three infection stage time points (24, 48 and 72 hpi) in isolate 88069. [file 12866_2014_308_MOESM7_ESM.tiff]

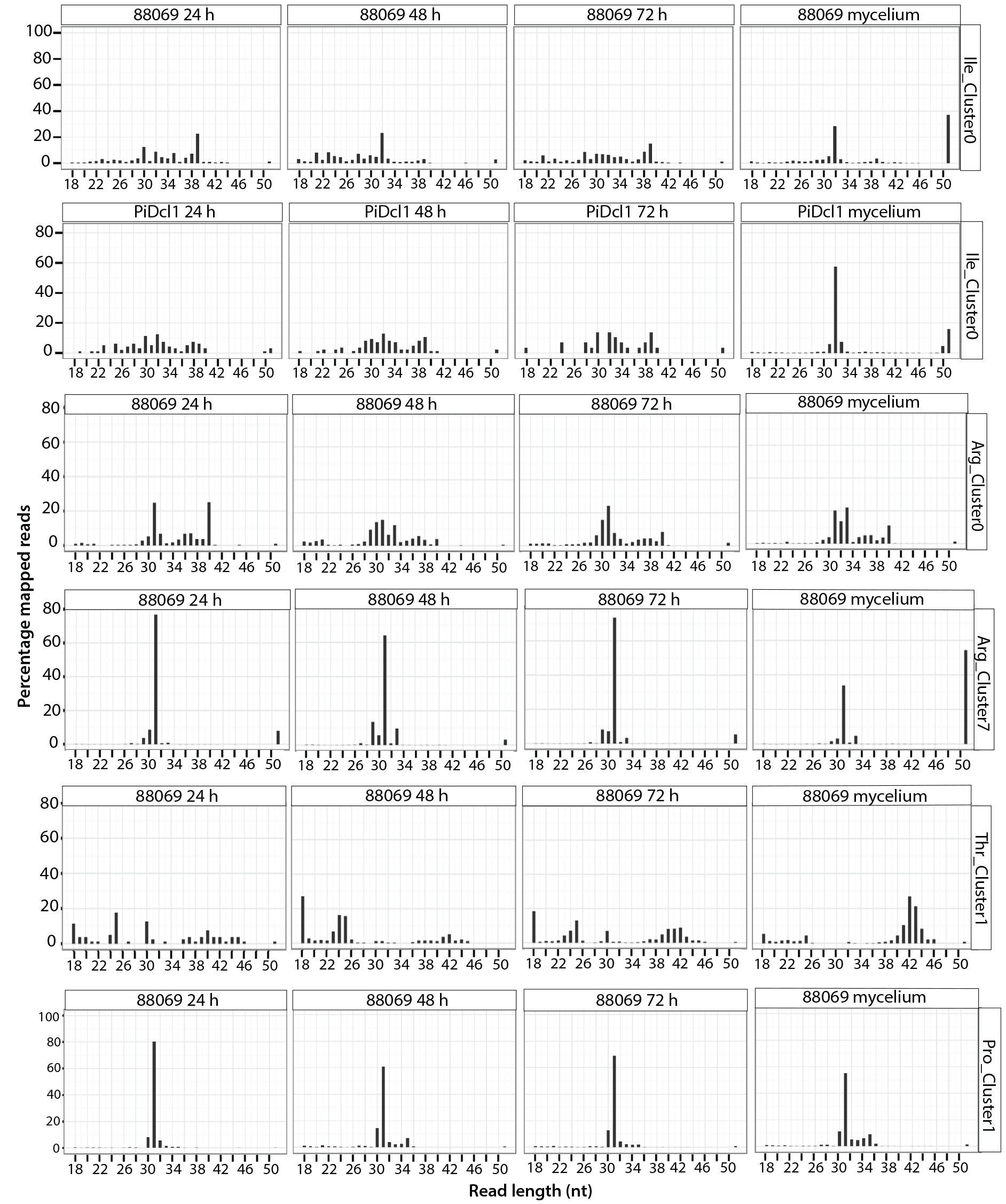

Supplement: Additional file 8: Figure S7. — Size distribution of Illumina sRNA reads mapping to individual tRNA clusters. sRNA sequence read lengths in the three infection stage libraries and the mycelium life cycle stage library in isolate 88069 and in the PiDcl1 silenced mutant. Y-axis: the percentage mapped sRNA reads of each length class. [file 12866_2014_308_MOESM8_ESM.tiff]

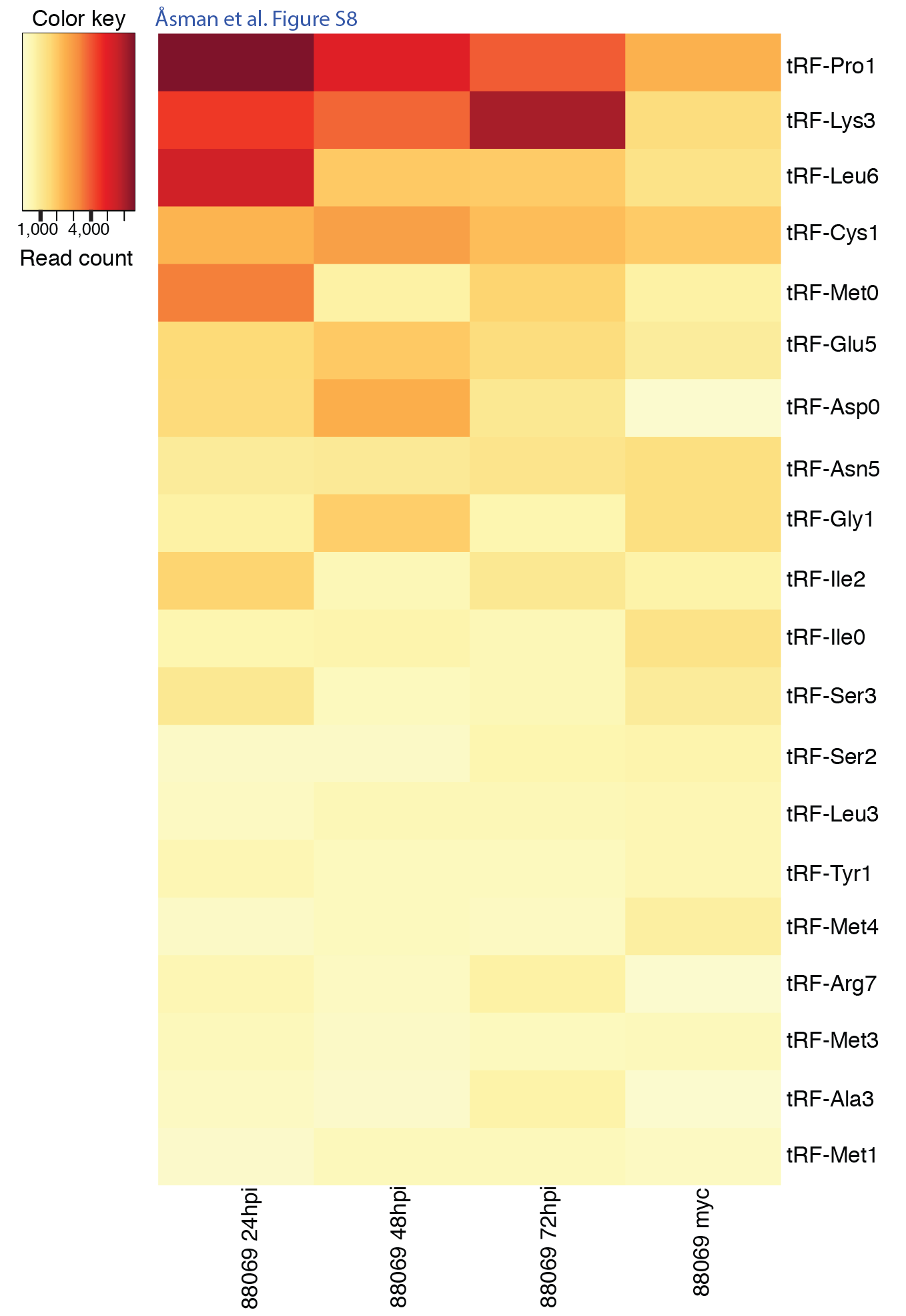

Supplement: Additional file 10: Figure S8. — Heatmap displaying tRNA-mapping read numbers. The top 20 tRFs exhibiting the highest numbers of normalized read counts across the four sequenced libraries in isolate 88069. 24, 48 and 72 hpi; three infection time points, myc; mycelium. Color key: normalized read counts. [file 12866_2014_308_MOESM10_ESM.tiff]

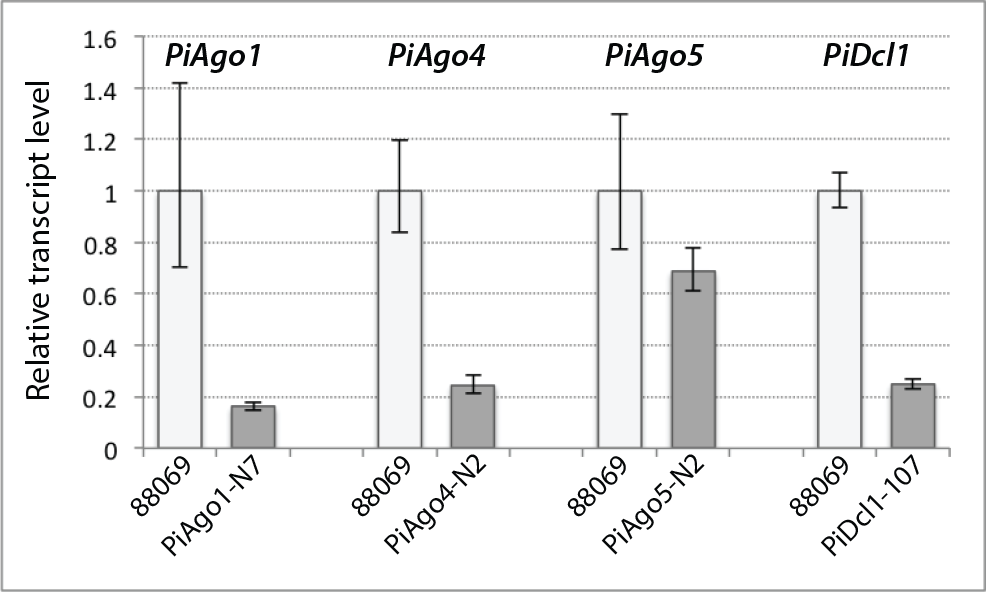

Supplement: Additional file 11: Figure S9. — Transcript levels in hairpin-transformed lines assayed by real-time RT-PCR. The four silenced lines used for Northern blot analysis of tRF levels. mRNA levels were normalized to the ActinA (PiActA) reference gene and are presented relative to the wild type isolate 88069 (assigned a value of 1.0). Total numbers of individual lines analyzed per gene: PiDcl1- 10, PiAgo1- 8, PiAgo4- 10 and PiAgo5- 6. Error bars represent confidence intervals from two technical replicates per sample in the PCR reaction. [file 12866_2014_308_MOESM11_ESM.tiff]
