# Supplementary material for: Fragmentation of tRNA in Phytophthora infestans asexual life cycle stages and during host plant infection
Source: BMC Microbiol. 2014 Dec 10;14:308. doi: 10.1186/s12866-014-0308-1 (PMC4272539; doi:10.1186/s12866-014-0308-1)
Supplement: Additional file 12: Table S3: — Sequences of oligonucleotides used as DNA probes. [file 12866_2014_308_MOESM12_ESM.docx]

**Table S3 Sequences of oligonucleotides used as DNA probes**

| **RNA identity** | **Oligo name** | **Sequence** | **Length (nt)** |
| --- | --- | --- | --- |
| Arg tRNA_cluster0 | Arg0-tRF  Arg0-tRFstar5p  Arg0-tRFstar3p | ACCCGCAATCTCCCGCTTC  CGGATGCCTTATCCATTGGGCCACGGGGAC  AGTCTCCGACGGGACTCGA | 19  30  19 |
| Ile tRNA_cluster0 | Ile0-tRF5 | ACTGTGCTCTACCGACTGAGCTATACGACC | 30 |
|  | Ile0-tRFstar3p | TGGTCATACAGGGGCTCGAACCCTGGACCG | 30 |
| Thr tRNA_cluster1 | Thr1-tRF5 | ATCGCCTTAACCACTCGGCCAAGCTGTC | 28 |
| Arg tRNA_cluster7 | Arg7-tRF5 | CCAGAGTGCTAACCATTACACCATAAGACC | 30 |
|  | Arg7-tRFstar3p | AGGTCCTACCGGGATTTAAACCCGGATTGC | 30 |
| Met tRNA_cluster3 | Met3-tRF5 | TTCCTCTGCGCCAAAGTGCT | 20 |
| Asn tRNA_cluster1 | Asn1-tRF5 | TCTACCGACTGAGCTAGACGGGT | 23 |
| Asp tRNA_cluster0 | Asp0-tRF5 | GTATACTAACCACTATACTAACGAGAA | 27 |
| Arg tRNA_cluster4 | Arg4-tRF5 | CTTGTGCTCTACCGACTGAGCTAGACGGGC | 30 |
| Gly tRNA_cluster0 | Gly0-tRF5 | GAGATATTTTACCACTAAACCAACGGCGC | 29 |
| Trp tRNA_cluster2 | Trp2-tRF5 | GTGCTCTACCACTGAGCTATTCCCCC | 26 |
| Leu tRNA_cluster0 | Leu0-tRF5 | CCCCTTAGACCACTCGGGCAAGATACC | 27 |
| Glu tRNA_cluster4 | Glu4-tRF5 | GATGAATCCTAACCATTAGACCAACGGTGC | 30 |
| Ser tRNA_cluster6 | Ser6-tRF5 | GCTCTACCACTGAGCTACCCGCGC | 24 |
| 5S rRNA | 5S | GCTTAACTTCACAGAGCAGAC | 21 |
